# Magnetic, Phonon and Optical Properties of Transition Metal and Rare Earth Ion Doped ZnS Nanoparticles

**DOI:** 10.3390/nano13010079

**Published:** 2022-12-23

**Authors:** Iliana Apostolova, Angel Apostolov, Julia Wesselinowa

**Affiliations:** 1 University of Forestry, Kl. Ohridsky Blvd. 10, 1756 Sofia, Bulgaria; 2 University of Architecture, Civil Engineering and Geodesy, Hristo Smirnenski Blvd. 1, 1046 Sofia, Bulgaria; 3 Sofia University “St. Kliment Ohridski”, J. Bouchier Blvd. 5, 1164 Sofia, Bulgaria

**Keywords:** ZnS nanoparticles, magnetization, coercive field, phonon energies, band gap, s-d model

## Abstract

The surface, size and ion doping effects on the magnetic, phonon and optical properties of ZnS nanoparticles are studied based on the s-d model including spin-phonon and Coulomb interaction, and using a Green’s function theory. The changes of the properties are explained on a microscopic level, due to the different radii between the doping and host ions, which cause different strains—compressive or tensile, and change the exchange interaction constants in our model. The magnetization increases with increasing small transition metal (TM) and rare earth (RE) doping concentration. For larger TM dopants the magnetization decreases. The phonon energies increase with increasing TM, whereas they decrease by RE ions. The phonon damping increases for all doping ions. The changes of the band gap energy with different ion doping concentration is also studied. Band gap changes in doped semiconductors could be due as a result of exchange, s-d, Coulomb and electron-phonon interactions. We have tried to clarify the discrepancies which are reported in the literature in the magnetization and the band gap energy.

## 1. Introduction

Zinc sulfide (ZnS) is one of the first semiconductors which have intrigued researches with its interesting fundamental properties and diverse applications, such as p-type conductors, catalyzators, field emitters, field effect transistors, UV-light sensors, chemical sensors (including gas sensors), biosensors, and nanogenerators [1]. Due to the remarkable size-dependent optical properties, ZnS nanoparticles (NPs) have many potential applications in ultraviolet light emitting diodes, flat panel displays, solar cells, optical sensors, photovoltaic cells, photocatalysts and many other optoelectronic devices [2,3,4,5,6,7,8]. For all these applications the nanostructures must have high dispersion, stability and size uniformity.

In the last years various studies have been made of room temperature ferromagnetism (RTFM) in pure ZnS nanoparticles (NPs), nanowires and thin films doped with TM (Ni, Mn, Co, Cr, Fe etc.) [9,10,11,12,13,14]. It must be noted that pure ZnS is diamagnetic. The RTFM in Fe-doped ZnS NPs was first reported by Sambasivam et al. [15]. Xie et al. [16] and Chen et al. [17] observed ferromagnetism in TM-doped ZnS by density functional theory (DFT) studies. Recently, Jindal and Sharma [18] investigated the magnetic and optical properties of TM and RE-doped ZnS NPs, and observed that both are ferromagnetic and show enhanced magnetization. Fe-doped ZnS NPs have the highest saturation magnetization value. By doping with RE Nd ions is found also an increase of the magnetization by Rao et al. [19]. However, in the reported results are some discrepancies. For example, Sambasivam et al. [20] observed a paramagnetic behavior and a decrease of *M* with increasing Co dopants in ZnS NPs. Li et al. [21] and Saikia et al. [22] showed paramagnetic behavior in Fe-doped ZnS NPs.

Moreover, there are many published optical studies of different ion-doped (Cu, Mn, Ni, Co, Sr, V, Ag etc.) ZnS nanostructures [23,24,25,26,27,28,29,30,31,32,33]. It is reported that the band gap $E_{g}$ increases with increasing TM doping concentration (Mn, Co, Ni, Cu, Ag, Cd) in ZnS NPs [30] and decreases with increasing Sr [31] or RE [34,35] dopants. It must be noted that there are observed opposite results, for example that $E_{g}$ decreases with increasing Mn–, Ni– and Co- doping in ZnS thin films [29] or with increasing Mn doping in ZnS NPs [36]. It can be seen that there are also some discrepancies. The optical and structural studies of ZnS NPs are reported by Mamiyev et al. [37]. It must be noted that there are also recent works in the field of other nanomaterials, such as PbTe, etc. and their optical applications [38,39].

The Raman spectroscopy is an important tool to study the structure and phase transitions of different materials. Raman studies show that compared with the Raman modes of undoped ZnS NPs the vibrational modes in ion-doped ZnS nanostructures are shifted towards lower or higher frequencies [32,40,41,42,43]. The origin for this shift is not clear.

However, magnetic, optical and phonon studies of ion-doped ZnS NPs based on a microscopic model have not been reported before. The origin of RTFM as well as the origin of the changes of different properties due to size and doping effects is still in debate. Moreover, we will clarify the reported discrepancies in some properties appearing by ion doping.

## 2. The Model and the Green’s Functions

A ZnS NP with a cubo-octahedral shape is observed by fixing a certain Zn spin in the center of the particle and all other spins are included into shells *n*, so that n=1 denotes the central spin and n=N—the surface shell.

The Hamiltonian which describes the TM or RE ion-doped ZnS NP is the s-d Hamiltonian:

(1) $$H_{s-d}=H_{sp}+H_{el}+H_{sp-el}.$$

$H_{sp}$ is the Heisenberg model of the localized spins:

(2) $$\begin{array}{ccc} H_{sp} & = & -\sum_{i,k}xJ_{ik}^{DI}S_{i}\cdot S_{k}-\sum_{i}D_{i}{\left(S_{i}^{z}\right)}^{2}\\ & - & \frac{1}{2}\sum_{i,j}F_{i,j}Q_{i}S_{j}^{z}-\frac{1}{4}\sum_{i,j,r}R_{i,j,r}Q_{i}Q_{j}S_{r}^{z}+h.c. \end{array}$$

where $S_{i}$ and $S_{i}^{z}$ are the spin-operators for the localized spins of the doping ions at site *i*, $J^{DI}$ is the exchange interaction between neighbouring sites *i* and *j* of the doping ions, *x* is the doping concentration, $D_{i}$ is the single-site anisotropy parameter. *F* and *R* are the spin-phonon interaction constants which are very important and must be taken into account. Due to surface effects the exchange interaction constants $J_{ij}=J\left(r_{i}-r_{j}\right)$ are different on the surface, denoted with the index *s*, and in the bulk, denoted with *b*.

$H_{el}$ represents the usual Hamiltonian of the conduction band electrons

(3) $$H_{el}=\sum_{ij\sigma }t_{ij}c_{i\sigma }^{+}c_{j\sigma }+\frac{1}{2}\sum_{ijkl,\sigma \sigma^{\prime }}v\left(ijkl\right)c_{i\sigma }^{+}c_{j\sigma^{\prime }}^{+}c_{k\sigma^{\prime }}c_{l\sigma }+i\sum_{ijk\sigma }A^{\prime }\left(ijk\right)c_{i\sigma }^{+}c_{j\sigma }\left(a_{k}^{+}-a_{k}\right).$$

$t_{ij}$ is the hopping integral, *v*—the Coulomb interaction, $A^{\prime }$—the electron-phonon interaction constant. $c_{i\sigma }^{+}$ and $c_{i\sigma }$ are Fermi-creation and -annihilation operators, *a*, $a^{+}$—the phonon annihilation and creation operators.

The operator $H_{sp-el}$ couples the two subsystems by an intra-atomic exchange interaction $I_{i}$:

(4) $$H_{sp-el}=\sum_{i}xI_{i}S_{i}s_{i}.$$

The spin operators $s_{i}$ of the conduction electrons at site *i* can be expressed as $s_{i}^{+}=c_{i+}^{+}c_{i-}$, $s_{i}^{z}=\left(c_{i+}^{+}c_{i+}-c_{i-}^{+}c_{i-}\right)/2$.

$H_{ph}$ contains the lattice vibrations:

(5) $$\begin{array}{ccc} H_{ph} & = & \frac{1}{2!}\sum_{i}{\left(\omega_{0i}\right)}^{2}a_{i}a_{i}^{+}+\frac{1}{3!}\sum_{i,j,r}B\left(i,j,r\right)Q_{i}Q_{j}Q_{r}\\ & + & \frac{1}{4!}\sum_{i,j,r,s}A\left(i,j,r,s\right)Q_{i}Q_{j}Q_{r}Q_{s}, \end{array}$$

where $Q_{i}$ and $\omega_{0i}$ are the normal coordinate and frequency of the lattice mode, respectively.

From the poles of the phonon Green’s function

(6) $$G_{ij}\left(t\right)=\langle \langle a_{i}\left(t\right);a_{j}^{+}\rangle \rangle$$

we obtain the phonon energy ω

(7) $$\begin{array}{ccc} \omega_{ij}^{2} & = & \omega_{0}^{2}-2\omega_{0}\left(\frac{1}{N^{\prime }}\sum_{k}A_{ijk}^{2}(\frac{{\bar{n}}_{i\sigma }-{\bar{n}}_{j\sigma }}{\epsilon_{ik\sigma }-\epsilon_{jk\sigma }-\omega_{0}}\right)+M_{i}M_{j}R_{ij}\delta_{ij}\\ & - & \frac{1}{2N^{\prime }}\sum_{r}A_{ijr}^{ph}\left(2{\bar{N}}_{r}+1\right)-B_{ij}^{ph}\langle Q_{ij}\rangle \delta_{ij}), \end{array}$$

with ${\bar{n}}_{i\sigma }=\langle c_{i\sigma }^{+}c_{i\sigma }^{-}\rangle$, ${\bar{N}}_{r}=\langle a_{r}^{+}a_{r}^{-}\rangle$ and the electronic energies

(8) $$\epsilon_{ij\sigma }=t_{ij}-\frac{\sigma }{2}I\langle S^{z}\rangle +\sum_{lm\sigma^{\prime }}v_{ilm}\left[{\bar{n}}_{l\sigma^{\prime }}\delta_{mj}-{\bar{n}}_{m\sigma^{\prime }}\delta_{lj}\delta_{\sigma \sigma^{\prime }}\right]+\sum_{k}A^{\prime }\left(ijk\right)\langle a_{k}^{+}-a_{k}\rangle .$$

The magnetization $M=\langle S^{z}\rangle$ of the TM- or RE-doped ZnS NPs is calculated from the Green’s function for the magnetic subsystem

(9) $${\tilde{G}}_{ij}=\ll S_{i}^{+};S_{j}^{-}\gg .$$

We obtain for arbitrary spin *S* value:

(10) $$M=\frac{1}{N}\sum_{i} [\left(S+0.5\right)\coth\left[\left(S+0.5\right)\beta E_{mi}\right)]-0.5\coth(0.5\beta E_{mi}],$$

where $E_{mi}$ are the spin excitations energies.

The band gap energy $E_{g}$ of ZnS NPs is defined by the difference between the valence and conduction bands with the electronic energies from Equation (8).

## 3. Results and Discussion

Bulk ZnS can crystallize in hexagonal wurtzite structure and cubic one. The hexagonal phase is metastable at temperatures lower than 1293 K, while the cubic phase remains stable at low temperatures [44]. With decreasing NP size, the relative stability of the two phases changes and low-temperature synthesis of small wurtzite ZnS NPs has been reported [45,46]. Phase control in the growth of ZnS crystals is important, because the phases have unique physical properties. For example, they show different lattice vibration properties and nonlinear optical coefficients [47].

**Figure 1:**
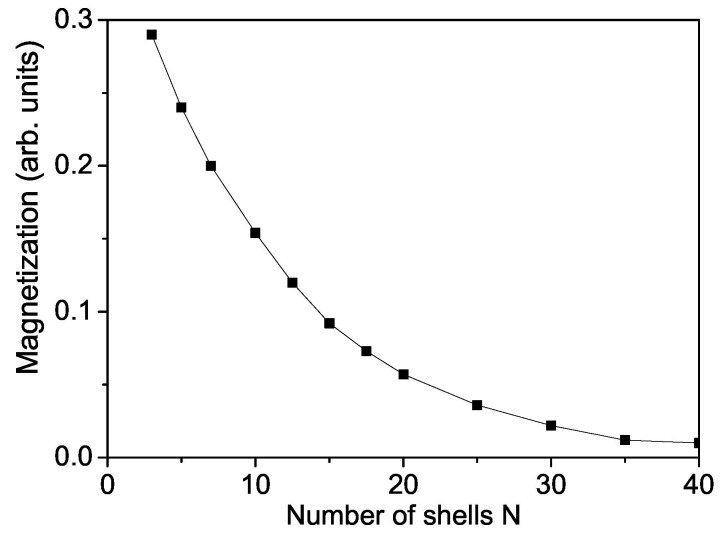
Size dependence of the spontaneous magnetization $M_{s}$ of a ZnS NP for *T* = 300 K and $J_{s}=1.2J_{b}$.

Firstly we will study the surface and size dependence of the spontaneous magnetization $M_{s}$ of a cubic ZnS NP. The numerical calculations are made using the relation $J_{s}>J_{b}$ taking into account the uncompensated spins which appear on the surface due to vacancies, impurities and surface defects. Let us emphasize that unpaired spins formed also by these defects play a role by the appearance of magnetism. Unpaired electrons exist when the compound has an odd number of electrons or because electron pairing is destabilized. This means that the more unpaired electrons, the stronger the magnetic property. It can be seen from Figure 1 that the spontaneous magnetization $M_{s}$ increases with decreasing NP size, i.e., ZnS NPs show ferromagnetism whereas the bulk compounds are diamagnetic. Below a critical size, $N_{cr}$ = 3 the ferromagnetism disappears, we have superparamagnetism [48]. Singh et al. [49] observed that ZnS nanomaterials show weak RTFM due to reduced dimensions, reduced coordination of atoms at the surface. Kumar et al. [50] reported also RTFM in ZnS nanopowders.

Now we will consider the magnetic behavior of magnetic ion-doped ZnS NPs which has received increasing interest belonging to spintronics. Doping of magnetic-ion may increase the Curie temperature that has great influence on spintronic devices. Thus, doped ZnS nanostructures become potential candidate with novel magnetic, electric and optical properties for spintronic and optoelectronic devices. By doping with TM ions which have different ionic radius in comparison with the host Zn ion there appear strains - compressive or tensile, which can modify the electronic and magnetic properties of the compound. Moreover, because the exchange interaction constant *J* depends on the lattice parameters, it can be different in the doped states $J_{d}$ compared to that in the undoped ones $J_{b}$.

**Figure 2:**
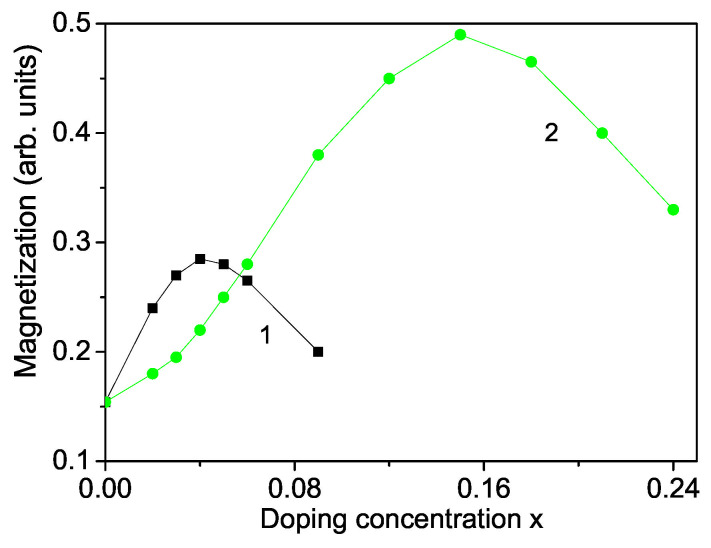
Doping concentration dependence of the spontaneous magnetization $M_{s}$ of a ZnS NP for *N* = 10 shells, *T* = 300 K for different doping ions: (1) Co; (2) Fe.

Let us analyze the concentration dependence of the spontaneous magnetization $M_{s}$ according to Equation (10). This is performed for a TM ion-doped ZnS NP with *N* = 10 shells. The results are presented in Figure 2 for Zn${}_{1-x}$Co${}_{x}$S and Zn${}_{1-x}$Fe${}_{x}$S for *T* = 300 K. The model parameters for Co-doped ZnS are: S = 3/2; *J* = 11.7 meV for x≤0.15, *J* = −11.7 meV for x>0.15, *I* = 0.2 eV, *v* = 0.4 eV; *D* = 4 meV; *F* = 22 cm${}^{-1}$, *R* = −18.5 cm${}^{-1}$, *B* = −2.9 cm${}^{-1}$, *A* = 6.6 cm${}^{-1}$, $J_{s}$ = 1.12$J_{b}$, $I_{s}$ = 1.12$I_{b}$. The values for the different exchange interaction constants *J* are estimated from the expression in mean-field theory $J=3k_{B}T_{C}/zS\left(S+1\right)$, where *z* is the number of nearest neighbors, *S*—the spin value, $T_{C}$—the critical temperature and $k_{B}$—the Boltzmann constant. From this relation we have obtained the exchange interaction constants of the bulk ZnS. The spin-phonon interaction constants *F* and *R* are determined from the Raman spectra at very low temperatures, taking two values at two different temperatures from the Raman phonon energy and solving the system of two equations with two unknown parameters, whereas the phonon-phonon interaction constants *A* and *B* - at temperatures above the Curie temperature $T_{C}$ (where the terms with *R* and *F* vanish). The ionic radius of Co${}^{3+}$ (0.65 $\dot{A}$) is less than that of Zn${}^{2+}$ (0.74 $\dot{A}$). From the experimental data [51] is seen that the lattice constant *a* decreases with increasing Co-concentration, *x* = 0–30%, i.e., there is a compressive strain. We have to chose the following relation $J_{d}>J_{b}$, $J_{d}\equiv J^{DI}$. Moreover, for low Co-concentrations the exchange interaction between the Co ions can be ferromagnetic due to the s-d interaction whereas for high Co dopants it can be antiferromagnetic due to the super-exchange Co–Co interaction. In Figure 2, curve 1, is presented the Co-concentration *x* dependence of $M_{s}$ for small *x* values. It follows the the *x*-dependence of the lattice parameters. Firstly, using $J_{d}>J_{b}$ the spontaneous magnetization $M_{s}$ increases and reaches a maximum at *x*∼ 0.04. For a higher Co concentration $M_{s}$ decreases and we have to chose $J_{d}<J_{b}$ for x>0.04. This is due to the antiferromagnetic super-exchange Co-Co interactions. A maximum ferromagnetic behavior was observed by Patel et al. [51] for 4 % Co-doped ZnS NPs. Let us emphasize that Kumar et al. [50] reported a minimum in the Co concentration dependence of the lattice constant *a* at *x*∼ 0.03 in ZnS NPs, which corresponds to the observed maximum in $M_{s}$. Let us emphasize that the spin-phonon interactions renormalize *J* to $J^{eff}=J+2F^{2}/\left(\omega_{0}-MR\right)$ and play an important role at low temperatures, because above $T_{C}$ the magnetization *M* vanishes and the temperature contribution to $J^{eff}$ vanishes, too. The anharmonic phonon–phonon interactions *A* and *B* are important at higher temperatures and mainly above the critical temperature $T_{C}$.

A similar behavior we obtain also for a Fe ion-doped ZnS NP (see Figure 2, curve 2) using the following model parameters: *S* = 5/2, *J* = 19.04 meV for x≤0.4, *J* = −19.04 meV for x>0.4, $J_{s}$ = 1.3$J_{b}$, *D* = 3 meV, *I* = 0.5 eV, $I_{s}$ = 1.3$I_{b}$, *v* = 0.5 eV, *F* = 23 cm${}^{-1}$, *R* = −20 cm${}^{-1}$, *B* = −3.1 cm${}^{-1}$, *A* = 7.05 cm${}^{-1}$. To investigate the observed RTFM in ZnS NPs we consider Zn${}_{1-x}$Fe${}_{x}$S NPs. It seems that smaller Fe${}^{3+}$ ions are incorporated into the Zn${}^{2+}$ sites of ZnS lattice, which do not change the wurtzite structure. The lattice parameters decrease with increasing Fe concentration [52]. The substitution of Zn${}^{2+}$ by Fe${}^{3+}$ leads to cation vacancies increasing because of charge variation, or by both Fe${}^{2+}$ and Fe${}^{3+}$ ions. The ionic radius of Fe${}^{2+}$(0.61 $\dot{A})$ or Fe${}^{3+}$(0.55 $\dot{A})$ is smaller than that of Zn${}^{2+}$(0.74 $\dot{A})$. The reduction of the lattice constants of Fe-doped ZnS NPs [14] leads in our microscopic model to the relation $J_{d}>J_{b}$, and to increasing of the magnetization *M* up to *x* = 0.15 (see Figure 2, curve 1). A similar increase of $M_{s}$ for small Fe dopants is observed experimentally by many Refs. [11,14,18]. The Fe${}^{3+}$ ion (*S* = 5/2) substitution of the Zn${}^{2+}$ host ions induces an extra magnetic moment. Thus, both, a ferromagnetic coupling between the doping Fe${}^{3+}$-ions and a carrier-mediated mechanism contribute additively to the increasing of $M_{s}$. For higher Fe doping concentration, x>0.15 due to the antiferromagnetic Fe-Fe interactions $M_{s}$ decreases. It could be noted that the maximum of the Fe-doped ZnS NP is higher that that of Co-doped one due to the higher spin value as well the larger exchange interaction constant. Kumar et al. [14] have observed also a maximum of $M_{s}$ in Fe-doped ZnS NPs at *x* = 2%, whereas Saikia et al. [22] at *x* = 10%. It must be noted that we observe a maximum in the spontaneous magnetization $M_{s}$ for Cr-doped ZnS NPs, too, in coincidence with the experimental data of Reddy et al. [53]. Such decrease in the spontaneous magnetization $M_{s}$ for higher dopant concentrations *x* has also been reported in ZnO nanostructures. Hu et al. [54] and Li et al. [21] have observed reduction of *M* with raising the Cr dopants in ZnO thin films.

**Figure 3:**
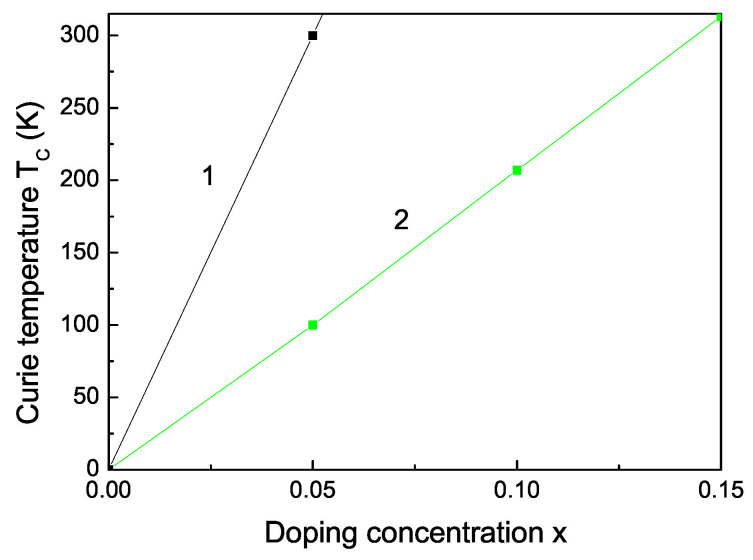
Co (1) and Fe (2) doping concentration dependence of the Curie temperature $T_{C}$ of bulk ZnS.

**Figure 4:**
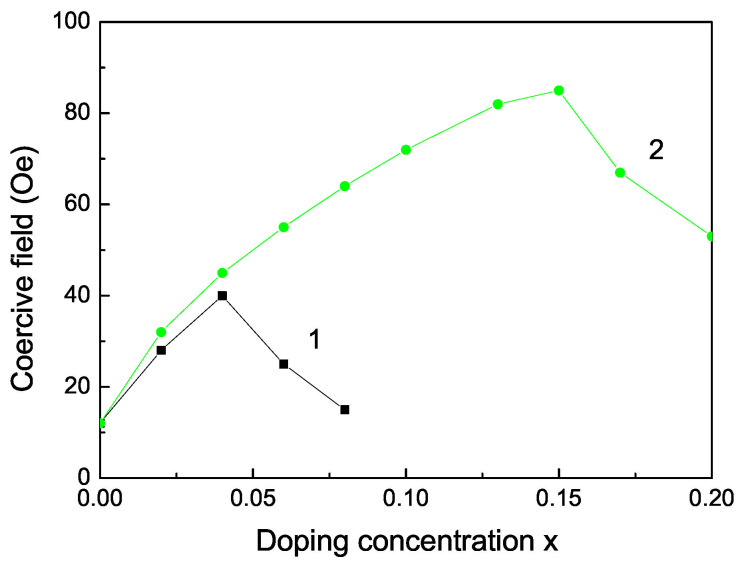
Doping concentration dependence of the coercive field of a ZnS NP for *N* = 10 shells, *T* = 300 K for different doping ions: (1) Co and (2) Fe ion doping.

The Curie temperature $T_{C}$ increases with increasing Fe doping concentration *x* (see Figure 3, curve 2) as experimentally observed [55]. This is mainly due to the p-d exchange interaction between the Fe 3d states and S 2p states. $T_{C}$ increases also with increasing Co dopants (see Figure 3, curve 1). Let us emphasize that we obtain a maximum also in the coercive field in dependence on the Fe doping concentration *x* (see Figure 4).

It must be noted that there are some discrepancies. Sambasivam et al. [15] Kumar et al. [14], Akhtar et al. [11] and Bhattacharya et al. [56] observed an increasing of $M_{s}$ for small Fe doping concentration in ZnS NPs, but Li et al. [21] and Saikia et al. [22] showed paramagnetic behavior in Fe doped ZnS NPs. Moreover, Sambasivam et al. [20] observed a paramagnetic behavior and a decrease of the spontaneous magnetization $M_{s}$ with increasing Co dopants in ZnS NPs.

**Figure 5:**
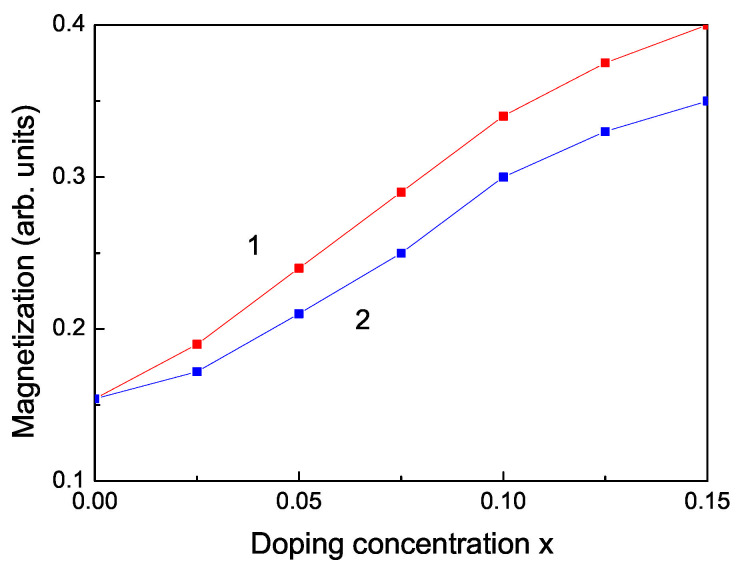
Doping concentration dependence of the spontaneous magnetization $M_{s}$ of a ZnS NP for *N* = 10 shells, *T* = 300 K for different doping RE ions: (1) Dy; (2) Nd.

We obtain RTFM and an increase of the spontaneous magnetization $M_{s}$ also by doping with RE ions, for example Dy, Nd, Tb. The ionic radii of Dy${}^{3+}$ and Nd${}^{3+}$ are 1.05 $\dot{A}$ and 1.12 $\dot{A}$, respectively, i.e., larger than the radius of the Zn${}^{2+}$ ion, i.e., we have a tensile strain. The lattice parameters increase with increasing the ion doping, as experimentally observed by Jindal et al. [18]. Therefore, the magnetization has to decrease. However, due to the strong ferromagnetic coupling between the RE ions and the strong s-d coupling we observe that the spontaneous magnetization $M_{s}$ increases (see Figure 5) in coincidence with the experimental data of [18,19].

**Figure 6:**
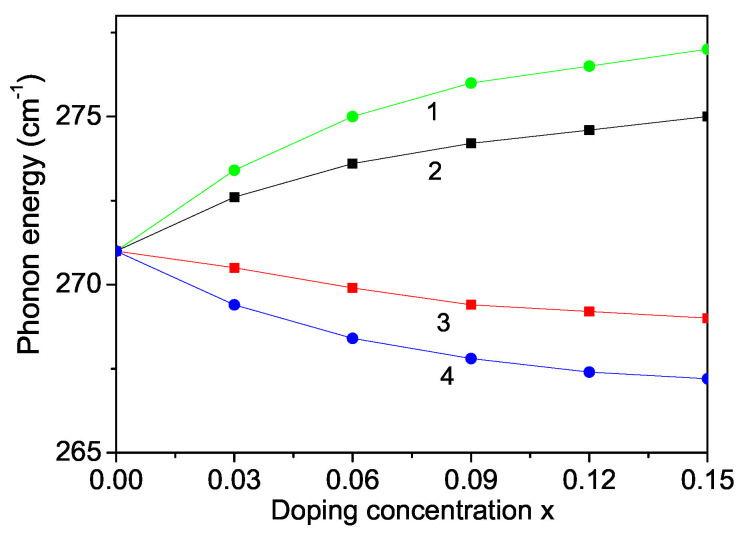
Doping concentration dependence of the phonon energy ω of a ZnS NP for *N* = 10 shells, *T* = 300 K and different doping ions: (1) Fe; (2) Co; (3) Dy; (4) Nd.

**Figure 7:**
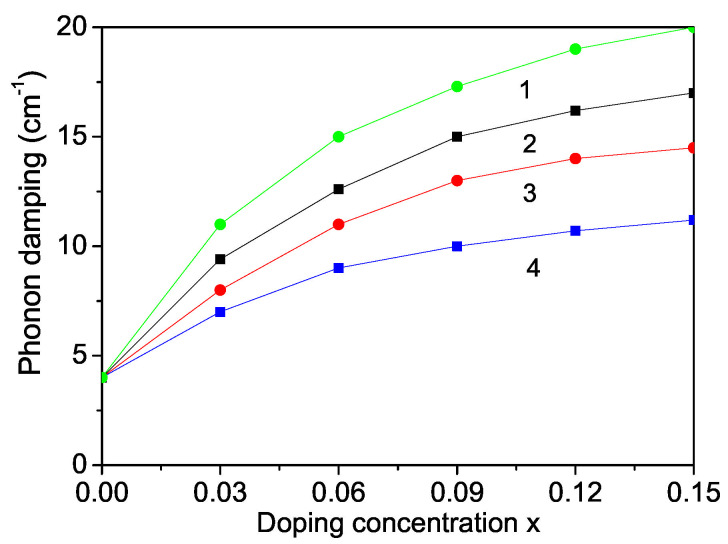
Doping concentration dependence of the phonon damping γ of a ZnS NP for *N* = 10 shells, *T* = 300 K and different doping ions: (1) Dy; (2) Nd, (3) Fe; (4) Co.

Raman scattering is a powerful probe to illustrate the effects on crystalline structures caused by ion incorporation. We will investigate now the phonon properties for pure and ion-doped ZnS NPs at room temperature. The line position and band width of the Raman spectra show changes with temperature, size and doping. The phonon energy shifts towards lower energy (271 cm${}^{-1}$) as compared to the bulk ZnS values (276 cm${}^{-1}$) and the phonon damping increases with decreasing NP size [32]. Surface optical phonons in ZnS nanostructures are observed by Ho et al. [57] and Kim et al. [40] using polarization dependent Raman scattering. We will now consider the doping effects on the phonon energy. The origin of these phonon changes is not clear at present. According to the discussion after Figure 2, due to the changes of the lattice parameters by the ion doping, due to the appearing compressive strain by doping with TM ions (Fe or Co) of ZnS NPs we chose additively to $J_{s}>J_{b}$ and $J_{d}>J_{b}$ the following spin-phonon interaction values $R_{d}>R_{b}$ which relation is valid also for the phonon–phonon interaction constants. The results are presented in Figure 6, curves 1 and 2. The phonon energy ω increases with increasing doping concentration in agreement with the experimental data of Fe-, Co- and Ni-doped ZnS NPs by Poornaprakash et al. [32]. However, considering the doping effects of RE ions (Dy or Nd), which ionic radius is much larger compared to that or the Zn ion, there appears a tensile strain, i.e., we have to chose the relations $J_{s}>J_{b}$, $J_{d}<J_{b}$, $R_{d}<R_{b}$. We observe a decrease of the phonon energy ω, see Figure 7, curves 3 and 4. A similar decrease of ω is reported in Sr-doped ZnS NPs by Boulkroune et al. [31], where the radius of the Sr ion is larger compared to that of the Zn ion, i.e., there appears a tensile strain similar to the RE ion. Harish et al. [41] have also observed a decrease of the transverse phonon mode with increasing Cu and Ce co-doping concentration.

We explained the doping effects leading to phonon energy changes on microscopic level, on the changes of the exchange interaction constants *J* due to the different ionic radii of the doping and host ions which lead to different strains, to changes of the lattice parameters. The anharmonic spin-phonon interaction which renormalizes the exchange interaction constant *J* to $J^{eff}=J+2F^{2}/\left(\omega_{0}-MR\right)$ (*M* is the magnetization, $\omega_{0}$ the unrenormalized phonon energy) plays an important role at low temperatures, whereas the phonon–phonon ones do so at higher temperatures. Moreover, the phonon energy is renormalized also due to the electron–phonon interaction $A^{\prime }$ which plays an important role at low temperatures. Therefore both electron–phonon and spin–phonon interactions must be taken into account.

The phonon damping which corresponds to the full width at half maximum of the Raman lines consists of additive contributions from the surface, bulk, spin–phonon and phonon–phonon interactions effects increases with increasing doping concentration for all dopants (see Figure 6).

**Figure 8:**
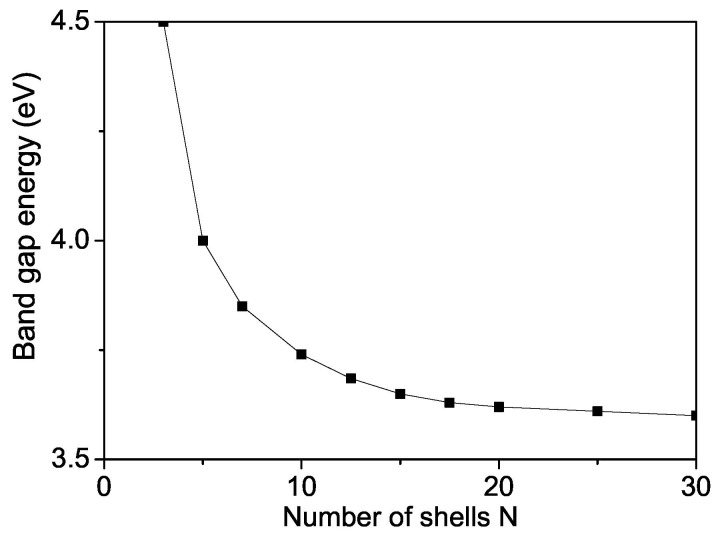
Size dependence of the band gap energy $E_{g}$ of a ZnS NP for *T* = 300 K.

The band gap energy $E_{g}$ for bulk ZnS varied from 3.54–3.68 eV [58,59,60]. We observed that $E_{g}$ value of a ZnS NP is 3.74 eV which is higher than the bulk one (see Figure 8) due to surface and size effects in coincidence with Refs. [18,54,60,61,62,63]. We will note that, from Equation (8), it can be seen $E_{g}$ decreases with increasing the s-d interaction *I* and increases with increasing the Coulomb interaction *v* and the electron-phonon interaction $A^{\prime }$, i.e., they are important and must be taken into account in order to observe correct results. There is some competition between all interactions.

The band gap of ZnS is 3.68 eV at room temperature. The band gap energy $E_{g}$ can be also modulated, can be reduced or enhanced by ion doping. On the basis of the discussion above we have calculated $E_{g}$ and observed that it decreases with increasing TM doping concentration where we have a compressive strain, for example Fe-, Co- doped ZnS NPs (see Figure 8, curves 1 and 2). We have considered the appeared strain as the mainly origin for the different properties of doped and undoped ZnS NPs. The strain changes the exchange interaction constants *J* which depend on the distance between the spins, on the lattice parameters, on the bonding angle, on the crystal and electronic structure. Thus taking into account in our microscopic model the s-d(f) interaction and the exchange interaction between the doping ions we have considered indirectly the electron structure, such as *f* orbital or *d* orbital occupation states as shown for example in Co-doped ZnS by some authors from first principle study [64,65]. The authors observed hybridisation of the 3D states of Co with the sp band of the host semiconductor. They have calculated the partial density of states of 3d-Co and 3p-S as function of energy and shown that in the energy from 0 to −5.0 eV below the Fermi level, d-orbitals of Co and p-orbital of S overlap, indicating the hybridization between S-p and Co-d orbitals. Our result is that a decrease of the band gap energy $E_{g}$ with increasing the Co dopants is in agreement with [29,51]. Let us emphasize that there are observed results contrary to our result that $E_{g}$ increases with increasing Fe or Co ion-doping in ZnS NPs [18]. The Fe or Co doping of ZnS is highly effective, reduces $E_{g}$ and can significantly improve the photocatalytic properties.

**Figure 9:**
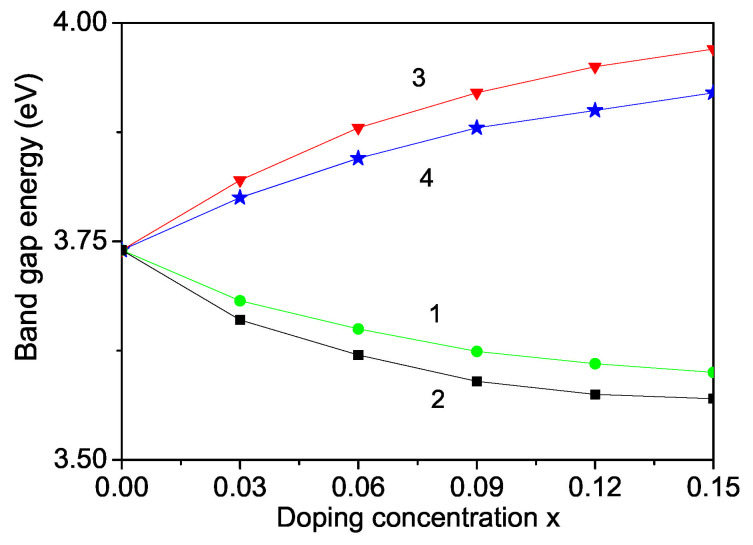
Doping concentration dependence of the band gap energy $E_{g}$ of a ZnS NP for *N* = 10 shells, *T* = 300 K and different doping ions: (1) Fe; (2) Co; (3) Y; (4) Mn.

By doping of ZnS NPs with ions which radius is larger than that of the Zn ion, for example Y${}^{3+}$ (*r* = 1.04 $\dot{A}$), we obtain that $E_{g}$ increases. The result is shown in Figure 9, curve 3, in agreement with [66]. Within our model we observe also an enhanced $E_{g}$ with increasing Mn${}^{2+}$ doping ions where the Mn ionic radius (0.83 $\dot{A}$) [30] is larger than that of the Zn${}^{2+}$ ion (0.74 $\dot{A}$) (see Figure 9, curve 4) in accordance with [67] but in disagreement with [23,30]. We would observe eventually a decrease of $E_{g}$ taking a strong s-d interaction.

Let us emphasize that in Figure 5 we have shown that the magnetization $M_{s}$ increases with increasing the RE doping ions Dy and Nd. This would lead to a decrease of the band gap energy $E_{g}$. Thus, this is consistent with the observed behavior, a decrease of the phonon energy ω with increasing RE dopants, see Figure 6, curves 3, 4.

## 4. Conclusions

In conclusion, the ion doping of ZnS NPs induces different strains and variation in the lattice parameters, which modifies the magnetic, optic and phonon properties. Using a microscopic model and the Green’s function theory we have studied the surface, size and TM and RE doping effects on these properties of ZnS NPs. The magnetization $M_{s}$ and the band gap energy $E_{g}$ increase with decreasing the NP size. $M_{s}$ and the Curie temperature $T_{C}$ increase for small TM and RE concentration. The realization of a high $T_{C}$ can be investigated for spintronic, magnetoelectronic or magnetooptical applications. The phonon energies increase with increasing TM dopants, whereas they decrease by RE ion doping. The phonon damping increases for all doping ions. The changes of the band gap energy $E_{g}$ with different ion doping concentration is also studied. Band gap changes in doped semiconductors could be due as a result of exchange, s-d, Coulomb and electron-phonon interactions. The band gap tuning behavior of ZnS NPs with different doping concentrations may find interesting applications in optoelectronic devices.

## Data Availability

Derived data supporting the findings of this study are available from the corresponding author upon reasonable request.
